# Supplementary material for: Dysfunctions of the paraventricular hypothalamic nucleus induce hypersomnia in mice
Source: eLife. 2021 Nov 17;10:e69909. doi: 10.7554/eLife.69909 (PMC8631797; doi:10.7554/eLife.69909)
Supplement: Figure 5—source data 1. [file elife-69909-fig5-data1.docx]

**Figure 5**—**source data 1.** Statistical analysis of whole-brain outputs from PVH^vglut2^ neurons**.**

| Regions | SUM | SNC | PB | ZI | NTS | SOX | PVT | ME | PAG | LSv | RSG | LA |
| --- | --- | --- | --- | --- | --- | --- | --- | --- | --- | --- | --- | --- |
| Proportion | ++ | + | ++ | + | ++ | + | ++ | ++ | ++ | + | ++ | +++ |

Note: The symbols in the table represent the proportion of nuclear-projection neurons in the whole brain projection, and the corresponding ranges are as follows: +++: > 10%; ++: 5% – 10%; +: < 5%.
